# Application of WST-8 based colorimetric NAD(P)H detection for quantitative dehydrogenase assays

**DOI:** 10.1186/s12858-019-0108-1

**Published:** 2019-04-08

**Authors:** Kamonwan Chamchoy, Danaya Pakotiprapha, Pornpan Pumirat, Ubolsree Leartsakulpanich, Usa Boonyuen

**Affiliations:** 1 Department of Molecular Tropical Medicine and Genetics, Faculty of Tropical Medicine, Mahidol University, Bangkok, 10400 Thailand; 2 Department of Biochemistry, Faculty of Science, Mahidol University, Bangkok, 10400 Thailand; 3 Center for Excellence in Protein and Enzyme Technology, Faculty of Science, Mahidol University, Bangkok, 10400 Thailand; 4 Department of Microbiology and Immunology, Faculty of Tropical Medicine, Mahidol University, Bangkok, 10400 Thailand; 5 National Center for Genetic Engineering and Biotechnology, National Science and Technology Development Agency, Pathumthani, 12120 Thailand

**Keywords:** WST-8, Tetrazolium salts, Dehydrogenase activity

## Abstract

**Background:**

The reduction of tetrazolium salts by NAD(P)H to formazan product has been widely used to determine the metabolic activity of cells, and as an indicator of cell viability. However, the application of a WST-8 based assay for the quantitative measurement of dehydrogenase enzyme activity has not been described before. In this study, we reported the application of an assay based on the tetrazolium salt WST-8 for the quantitative measurement of dehydrogenase activity. The assay is performed in a microplate format, where a single endpoint is measured at 450 nm.

**Results:**

The optimized dehydrogenase-WST-8 assay conditions, the limit of detection (LOD), accuracy, and precision for measuring NAD(P)H, were demonstrated. The sensitivity of the WST-8 assay for detecting NAD(P)H was 5-fold greater than the spectrophotometric measurement of NAD(P)H absorption at 340 nm (LOD of 0.3 nmole vs 1.7 nmole, respectively). In the dehydrogenase assay, the colorimetric WST-8 method exhibits excellent assay reproducibility with a Z’ factor of 0.9. The WST-8 assay was also used to determine dehydrogenase activity in biological samples, and for screening the substrate of uncharacterized short-chain dehydrogenase/oxidoreductase from *Burkholderia pseudomallei.*

**Conclusion:**

The results suggest that the WST-8 assay is a sensitive and rapid method for determining NAD(P)H concentration and dehydrogenase enzyme activity, which can be further applied for the high-throughput screening of dehydrogenases.

## Background

Nicotinamide adenine dinucleotide (NAD^+^/NADH) and nicotinamide adenine dinucleotide phosphate (NADP^+^/NADPH) are important biological molecules serving as cofactors in various enzymatic reactions essential for cellular metabolism, mitochondrial functions, protection against oxidative stress, signal transduction, and cell death [1–3]. Various methods are used to assay NAD(P)H [4–6]. Both NADH and NADPH can absorb light at 340 nm and have intrinsic fluorescence. Therefore, the activity of enzymes producing or consuming NAD(P)H (dehydrogenases and oxidoreductases) is commonly determined by measuring the absorbance of NAD(P)H at 340 nm, or monitoring fluorescence [7–9]. Though the detection of NAD(P)H absorption or fluorescence is useful for characterizing enzymes, occasionally, the method is not sufficiently sensitive and/or specific for measuring low concentrations of NAD(P)H. While electrochemical methods for detecting NAD(P)H, such as modified graphene and glassy carbon electrodes, are very attractive, their application in dehydrogenase activity assay remains limited [10, 11]. Electrochemical methods are expensive and require experienced laboratory technicians. In addition, particular substances could be absorbed on the electrode surface and interfere with the assay. Direct detection by oxidation of NADH usually results in a high potential at the platinum or carbon bare electrode. The oxidized form (NAD^+^) shows strong absorption on the electrode, causing fouling, and leading to reduced sensitivity, reproducibility, and stability [12]. Moreover, redox active species, such as ascorbic acid, uric acid, and glucose, reportedly interfere with the detection of NADH [13]. The bioluminescent assay is very sensitive for NAD(P)H detection but is quite expensive [14].

A colorimetric method using tetrazolium salts for the measurement of NAD(P)H concentration has been reported [15, 16]. Although tetrazolium salts have been used widely in biological and clinical studies, their application is limited by the low solubility of the formazan product [17–20]. Recently, several water-soluble tetrazolium salts (WST), which include WST-1, WST-3, WST-4, WST-5, WST-8 [2-(2-methoxy-4-nitrophenyl)-3-(4-nitrophenyl)-5-(2,4-disulfophenyl)-2H-tetrazolium, monosodium salt], and WST-9, have been synthesized [21, 22]. In the presence of an electron mediator, such as 1-methoxy-5-methylphenazium methylsulfate (1-mPMS), WST is readily reduced by NAD(P)H to produce a formazan product, which can be determined by monitoring absorbance in the range 430–550 nm. The absorbance of formazan is proportional to the NAD(P)H concentration, so that the WST-based colorimetric assay [21] shows potential for various qualitative and quantitative applications.

**Scheme 1:**
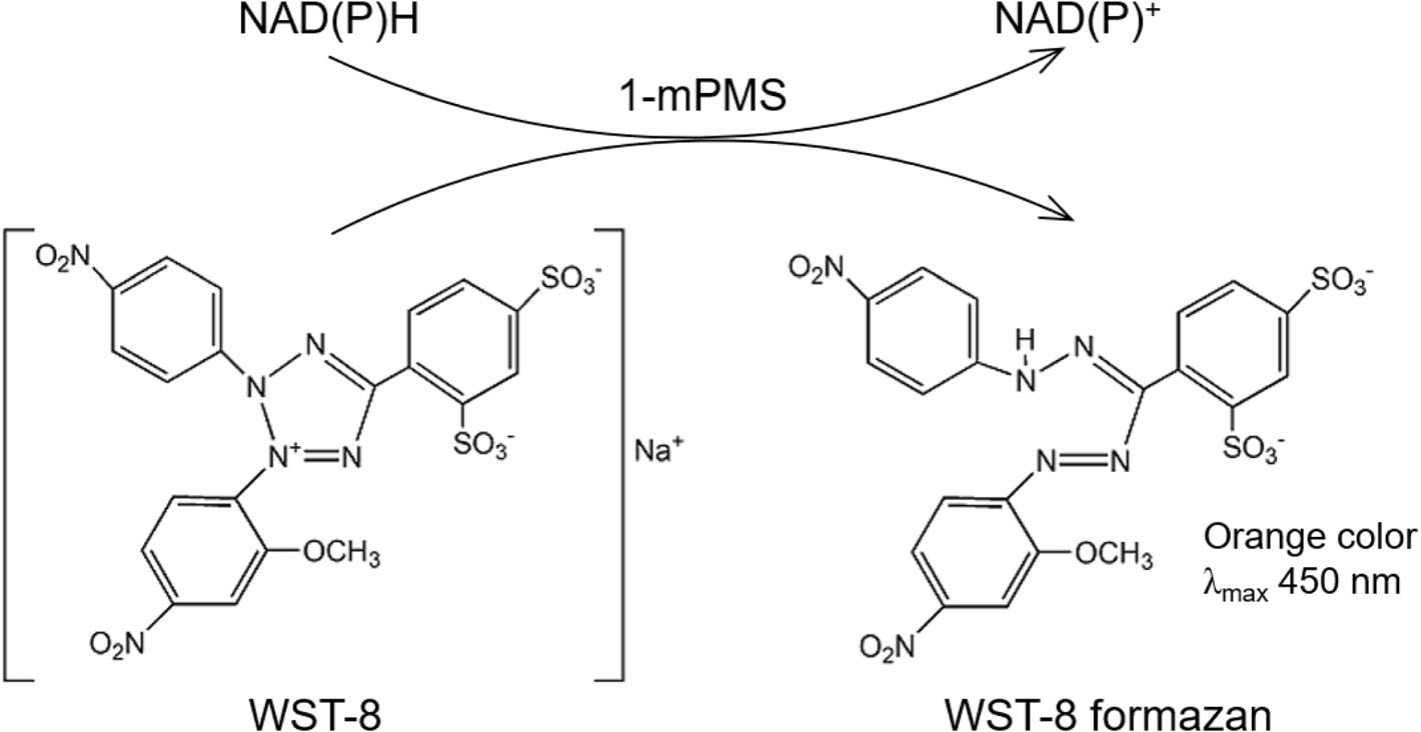
Schematic representation of the reaction for detection of dehydrogenase activity using colorimetric WST-8 assay

WST-8 is commercially available in the form WST-8/1-mPMS (Dojindo). It is among one of the most popular tetrazolium salts used as the NAD(P)H detection method in cell proliferation and enzymatic assays. WST-8 is reduced by NAD(P)H to produce the formazan product, a strong orange dye with maximum absorption at 450 nm (Scheme 1). The WST-8 method exhibits greater sensitivity and efficiency for measuring bacterial viability and antimicrobial susceptibility compared with the standard broth microdilution method [23–25]. Moreover, the WST-8 assay is more sensitive than WST-1, especially at neutral pH [21]. Therefore, the WST-8 colorimetric method is useful for the rapid determination of NAD(P)H and could be a valuable tool for screening cell viability and cytotoxicity [26–28]. WST-8 was used in the qualitative measurement of glucose-6-phosphate dehydrogenase (G6PD) activity. Screening for G6PD deficiency using the WST-8 method was shown to be sensitive and highly specific compared with the commercial rapid diagnostic test [29]. While WST-8 is commonly used in qualitative studies, there have been very few reports of its application in quantitative assays [29–31]. Furthermore, quantitative measurement of glucose dehydrogenase (GDH) activity using WST-8 has not been previously described. Though the commercial GDH assay kit is widely available, it is costly and therefore unsuitable for high-throughput screening.

In this study, the WST-8 colorimetric method was applied to quantify the concentration of NAD(P)H and the enzymatic activity of NAD(P)^+^-dependent dehydrogenases. GDH and G6PD were studied as models to evaluate the performance of the WST-8-based enzymatic assay. The assay conditions, which include the effect of pH, the concentrations of WST-8, cofactor and substrate, and reaction-time course, were optimized. The optimized conditions were used to measure the dehydrogenase activity of purified enzymes, as well as dehydrogenase activity in biological samples. The efficiency of the method developed was compared with that of the conventional UV-spectrophotometric method, where absorbance at 340 nm of NAD(P)H was measured. Furthermore, the assay was applied in substrate screening of an uncharacterized short-chain dehydrogenase/oxidoreductase (SDR) from *Burkholderia pseudomallei.* It was demonstrated that the WST-8 assay is rapid and sensitive, and can be used for dehydrogenases high-throughput screening.

## Methods

### Materials

A Cell Counting kit-8 containing 5 mM WST-8 and 0.2 mM 1-mPMS was obtained from Sigma-Aldrich (St. Louis, MO, USA). NAD(P)^+^, NAD(P) H, D-(+)-glucose, and glucose-6-phosphate (G6P) were purchased from Sigma-Aldrich (St. Louis, MO, USA). Other chemicals were purchased from Merck (Darmstadt, Germany). *Bacillus* GDH was obtained from a GDH activity colorimetric assay kit (Biovision, Mountain View, USA).

### G6PD expression and purification

The human G6PD enzyme was expressed and purified as previously described [32]. In brief, the enzyme was expressed in *E. coli* BL21 (DE3). The cells were grown at 37 °C until OD_600_ reached 0.8; protein expression was induced with 1 mM isopropyl β-D-thiogalactoside (IPTG). The cells were grown at 20 °C for an additional 20 h and harvested by centrifugation. The cell pellet was resuspended in lysis buffer (20 mM sodium phosphate pH 7.4, 300 mM NaCl, 10 mM imidazole) and lysed by sonication. After centrifugation at 20,000 *x* g for 1 h, the supernatant was incubated with cobalt TALON Metal Affinity Resin (BD Biosciences). Unbound proteins were removed by washing buffer (20 mM sodium phosphate pH 7.4, 300 mM NaCl, 20 mM imidazole) and G6PD protein eluted with increasing imidazole concentrations from 40 to 400 mM in 20 mM sodium phosphate pH 7.4, 300 mM NaCl. The G6PD protein was dialyzed against 20 mM Tris-HCl pH 8.0 containing 10% glycerol (*v*/v) and stored at − 20 °C. The purity of the protein was visualized by sodium dodecyl sulfate-polyacrylamide gel electrophoresis (SDS-PAGE) and the protein concentration was determined by Bradford assay [33].

### Microplate assay

#### Optimization of enzymatic assay using WST-8

The WST-8 assay was performed in a 96-well plate (Costar, Corning, NY, USA) with a total volume of 150 μL. The reaction conditions—buffer pH, incubation time, and the concentrations of WST-8, substrate and NAD(P)^+^—were optimized for determining GDH and G6PD activities. The assays were performed at 37 °C and 25 °C for GDH and G6PD, respectively. Absorbance at 450 nm was measured with the reference wavelength at 600 nm using a microplate reader (Sunrise, Tecan, Männedorf, Switzerland). Absorbance at 450 nm of a reaction mixture set up in the absence of substrate was used for background subtraction.

For the effect of pH, the activities of *Bacillus* GDH and human G6PD were determined in a buffer mixture containing 20 mM of each of the following buffers: MES [2-(*N*-morpholino) ethanesulfonic acid], MOPS [3-(*N*-morpholino) propanesulfonic acid], HEPES [4-(2-hydroxyethyl)-1-piperazineethanesulfonic acid], Tris-HCl, and CAPSO ***[****3-(cyclohexylamino)-2-hydroxy-1-propanesulfonic acid]*, at pH range 6.0–9.5. Incubation time was varied from 0 to 120 min for GDH and 0 to 15 min for G6PD. The concentration of WST-8 was varied between 0 and 300 μM. For the GDH assay, the concentrations of glucose substrate and NAD^+^ were 0–500 mM and 0–1 mM, respectively. For the G6PD assay, the concentrations of the NADP^+^ substrate and G6P were varied from 0 to 400 μM and 0–1 mM, respectively.

#### NADH and NADPH standard curve

The linear response curve for formazan absorbance (450 nm) at varying NAD(P)H concentrations was constructed. NADH and NADPH were treated with WST-8. The total volume of 150 μL contained 20 mM Tris-HCl (pH 9.0 for NADH and pH 8.0 for NADPH), 200 μM WST-8/8 μM 1-mPMS, and varying amounts of NAD(P)H (0–33 nmole). Formazan absorbance at 450 nm was plotted versus the amount of NAD(P)H and the linear range with a correlation coefficient > 0.99 was selected [34].

#### Accuracy and precision determination

To determine the accuracy and precision, known NAD(P)H amounts within the linear range were measured on five consecutive days in triplicate. NADH amounts were 4.3, 8.5, and 12.8 nmole, and NADPH amounts were 4.3, 8.6, and 12.9 nmole. The accuracy of the assay was expressed as the percent relative error (% RE), where % RE = 100% × (measured amount - prepared amount)/prepared amount. Both within- and between-run precisions were assessed as percent coefficient of variation (%CV).

#### Enzyme activity assay

GDH and G6PD activities were determined according to the optimal conditions obtained. The assays were performed at 37 °C and 25 °C for GDH and G6PD, respectively. For the GDH assay, the standard reaction mixture contained 20 mM Tris-HCl pH 9.0, 250 mM glucose, 1 mM NAD^+^, 200 μM WST-8/8 μM 1-mPMS and 1 μg GDH enzyme (Biovision, Mountain View, USA). For the G6PD assay, the standard reaction mixture contained 20 mM Tris-HCl pH 8.0, 10 mM MgCl_2_, 500 μM G6P, 200 μM NADP^+^, 200 μM tetrazolium salt and 0.1 μg G6PD enzyme. The amount of NAD(P)H generated was determined using the standard curve described above. Enzyme activity was expressed as nanomole of NAD(P)H produced per minute per microgram of protein (nmole/min/μg).

#### Determination of steady-state kinetic parameters for GDH and G6PD

To further confirm the performance and accuracy of the dehydrogenase assay using WST-8, the kinetic parameters for GDH and G6PD were determined and compared with those obtained from the UV-spectrophotometric standard method, which measures the absorbance of NAD(P)H at 340 nm [32, 35–37]. The assays were performed at 37 °C and 25 °C for GDH and G6PD, respectively. For the GDH enzyme, to determine the *K*_*M*_ for glucose, the assay was performed by fixing the concentration of NAD^+^ at 1 mM and varying the concentrations of glucose from 0 to 500 mM, while the *K*_*M*_ for NAD^+^ was determined by fixing the concentration of glucose at 250 mM and varying the concentrations of NAD^+^ from 0 to 1 mM. For the G6PD enzyme, to determine the *K*_*M*_ for the G6P substrate, the G6P concentration was varied from 0 to 500 μM, while fixing the concentration of NADP^+^ at 200 μM. To determine *K*_*M*_ for NADP^+^, the concentration of NADP^+^ was varied from 0 to 200 μM, while fixing the concentration of G6P at 500 μM. The rate of NAD(P)H product formation was calculated and expressed as micromolar NAD(P)H produced per minute (μM/min). The kinetic parameters were determined by fitting the data to the Michaelis–Menten equation using GraphPad Prism (GraphPad Software).

#### Applications of the WST-8 assay

The WST-8 assay was applied to measure the dehydrogenase activity of the biological samples. G6PD activity in *E. coli* crude extract was measured at 25 °C. Crude extracts (20 μg) of *E. coli* harboring empty pET28a and recombinant pET28a-G6PD plasmids were assayed in the standard reaction mixture. The absorbance obtained from *E. coli* crude extracts harboring recombinant pET28a-G6PD plasmid was subtracted with that of *E. coli* crude extract harboring empty pET28a plasmid. Enzyme activity was determined using NADPH standard curve and was expressed as nanomole of NADPH produced per minute per microgram of protein (nmole/min/μg).

The WST-8 assay was also used to screen for the substrate of an uncharacterized SDR from *B. pseudomallei*. Crude lysate (150 μg) of *E. coli* expressing recombinant SDR was screened for dehydrogenase activity using various substrates, including sugars, alcohols, and aldehydes. Reactions were performed at 37 °C in the standard reaction mixtures containing 20 mM Tris-HCl pH 8.0, 200 μM tetrazolium salt, 500 μM NAD(P)^+^ and substrate (concentration ranges from 50 mM to 250 mM). The enzyme activity of *E. coli* lysate expressing recombinant SDR was subtracted with *E. coli* lysate without SDR. The activity of SDR towards different substrates was expressed as nanomolar of NAD(P)H produced per minute (nM/min).

#### Spike recovery

One microgram of GDH or 0.2 μg of G6PD was spiked into 1.5 μL of fetal bovine serum (FBS). The dehydrogenase activity of the spiked samples was monitored at 450 nm in triplicate. The rate of NAD(P)H production measured from the spiked samples was subtracted with that from the FBS. The %recovery = 100% x (measured rate/expected rate).

#### Assay interference of WST-8/1-mPMS system

To determine assay interference in the absence of NAD(P)H, 150 μL reaction mixtures containing 20 mM Tris-HCl pH 8.0 and 200 μM WST-8 were mixed with various chemicals, including mono- and di-saccharides (250 mM), phosphorylated sugars (1 mM), alcohols (100 mM), aldehydes (50 mM), NAD(P)^+^ (1 mM), detergents (0–1%) and reducing agents (0–10 mM). Absorbance at 450 nm was followed at 37 °C to evaluate assay interference.

### UV-spectrophotometric assay

#### Enzyme activity assay

To validate the performance of the WST-8 assay, the conventional UV-spectrophotometric method (measurement of NAD(P)H absorption at 340 nm) was used as the standard method for dehydrogenase assay. The assays were performed at 37 °C and 25 °C for GDH and G6PD, respectively. For the GDH assay, the 1 mL reaction mixture contained 20 mM Tris-HCl pH 9.0, 250 mM glucose, 1 mM NAD^+^, and 5 μg GDH enzyme. For the G6PD assay, the reaction mixture contained 20 mM Tris-HCl pH 8.0, 10 mM MgCl_2_, 500 μM G6P, 200 μM NADP^+^, and 0.5 μg G6PD enzyme. The reaction was initiated with the addition of the enzyme and the rate of reaction was monitored at 340 nm for 30 and 10 min for GDH and G6PD, respectively. Blank reactions were carried out in the absence of glucose substrate. The reactions were monitored using a UV-2700 UV-VIS spectrophotometer (Shimadzu). The amount of NAD(P)H generated was calculated using molar extinction coefficient 6220 M^− 1^ cm^− 1^ for NAD(P)H. Enzyme activity was expressed as nanomole of NAD(P)H produced per minute per microgram of protein (nmole/min/μg).

#### NADH and NADPH standard curve

To determine the limit of detection of the conventional UV-spectrophotometric method, the NAD(P)H standard curve was constructed. The total volume of 1 mL contained 20 mM Tris-HCl (pH 9.0 for NADH and 8.0 for NADPH), and various amounts of NAD(P)H (0–860 nmole). Absorbance at 340 nm was monitored.

#### Determination of accuracy and precision

The amounts of NADH were 102, 212.5, and 340 nmole; and the amounts of NADPH were 103.2, 215, and 344 nmole. The within- and between-run accuracy and precision of the assay were calculated as described above.

#### Measurement of G6PD activity in biological samples

G6PD activity in *E. coli* crude extract was determined by assaying crude extract (100 μg) of *E. coli* harboring recombinant pET28a-G6PD plasmid with the reaction mixture mentioned above. Enzyme activity was determined using molar extinction coefficient 6220 M^− 1^ cm^− 1^ and was expressed as nanomole of NADPH produced per minute per microgram of protein (nmole/min/μg).

### Statistical analysis

All experiments were performed in triplicate. All analyses were performed with GraphPad Prism software and the results presented as mean ± standard deviation.

## Results

### Optimization of GDH and G6PD assay using WST-8

**Fig. 1:**
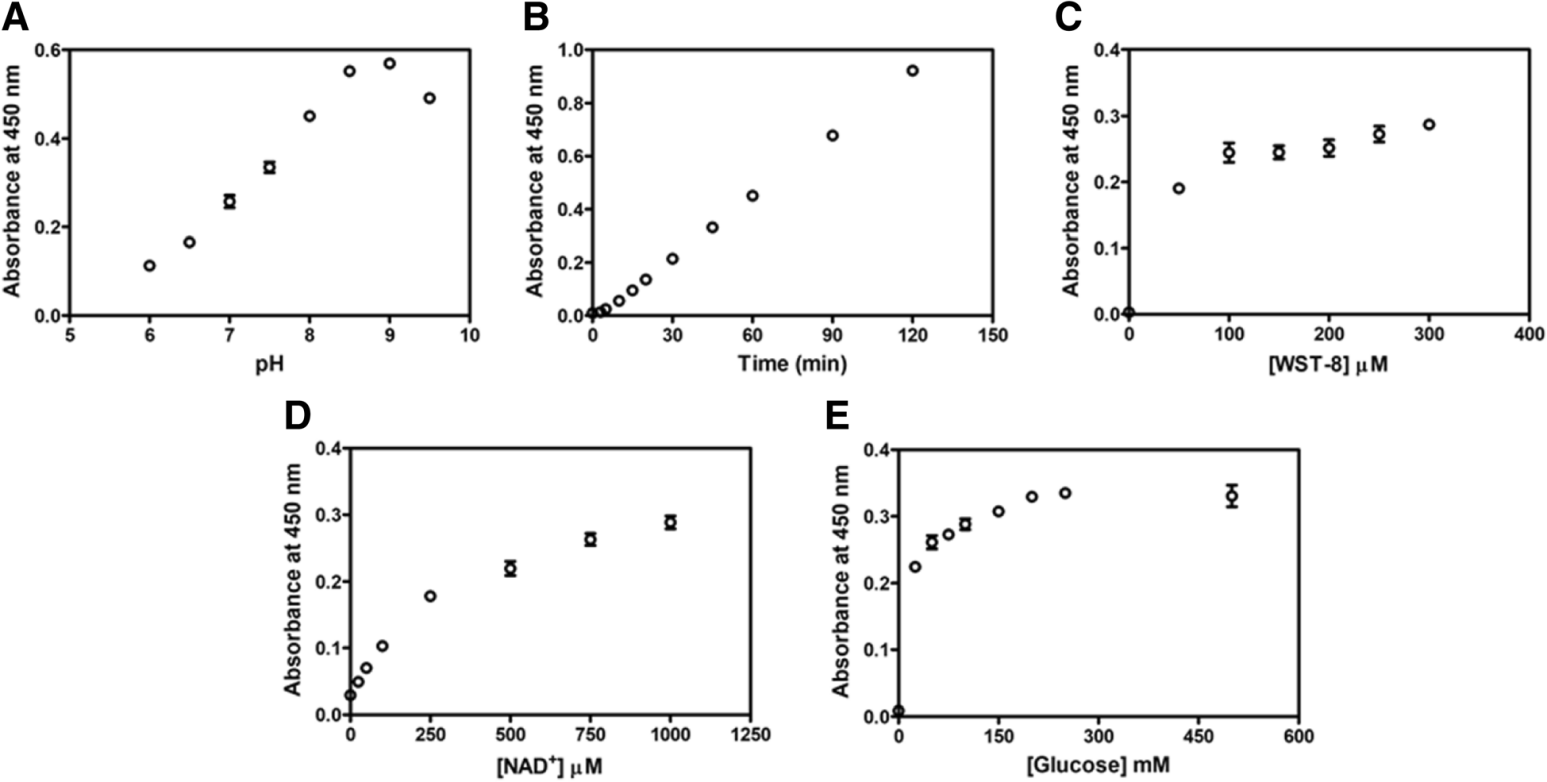
Optimization of GDH assay conditions using colorimetric WST-8. **a** The effect of pH on enzymatic reaction. **b** Reaction time course of the assay. **c** The effect of WST-8/1-mPMS on the absorbance. **d** The effect of NAD^+^ concentration on the enzymatic assay. **e** The effect of glucose concentration on the enzymatic assay

The assay conditions for the GDH and G6PD reactions were optimized. The pH effect was elucidated in a buffer mixture containing 20 mM each of MES, MOPS, HEPES, Tris-HCl and CAPSO (pH range 6.0 to 9.5). The result is shown in Fig. 1a. *Bacillus* GDH exhibited maximum activity at pH 9.0, which accords well with a previous report [35]. To optimize incubation time, GDH activity was monitored every 5 min. A linear correlation between absorbance at 450 nm and incubation time was observed for up to 120 min (Fig. 1b). An incubation time of 60 min was selected for further studies. For the concentration of WST-8, the absorbance at 450 nm increased with WST-8 concentration until the concentration of WST-8 reached 100 μM (Fig. 1c). This demonstrated that a minimum concentration of 100 μM of WST-8 was required in the GDH assay. Our results suggested that the concentration of WST-8 could be minimized for purposes of economy; however, at least 100 μM of WST-8 is required in the reaction to warrant the optimal sensitivity of the assay. To ensure that the concentration of WST-8 present is not limited for the assay and maximum sensitivity is reached, 200 μM of WST-8 was included in the standard reaction mixture for further experiments. Previous studies reported the use of 125 to 250 μM WST-8 for G6PD activity assay for G6PD deficiency screening [38, 39]. It is worth mentioning that the concentrations of WST-8 and 1-mPMS could not be optimized individually as WST-8 is commercially available only in the form of a mixture with 1-mPMS. Next, the concentrations of glucose and NAD^+^ substrates were optimized for GDH activity assay using WST-8. Absorbance at 450 nm increased with NAD^+^ concentration and reached a plateau at around 500 μM (Fig. 1d). For the glucose substrate, no significant increase in absorbance at 450 nm was observed when the glucose concentration was greater than 150 mM (Fig. 1e). Sufficient glucose (250 mM) and NAD^+^ (1 mM) were therefore included in the standard reaction mixture.

**Fig. 2:**
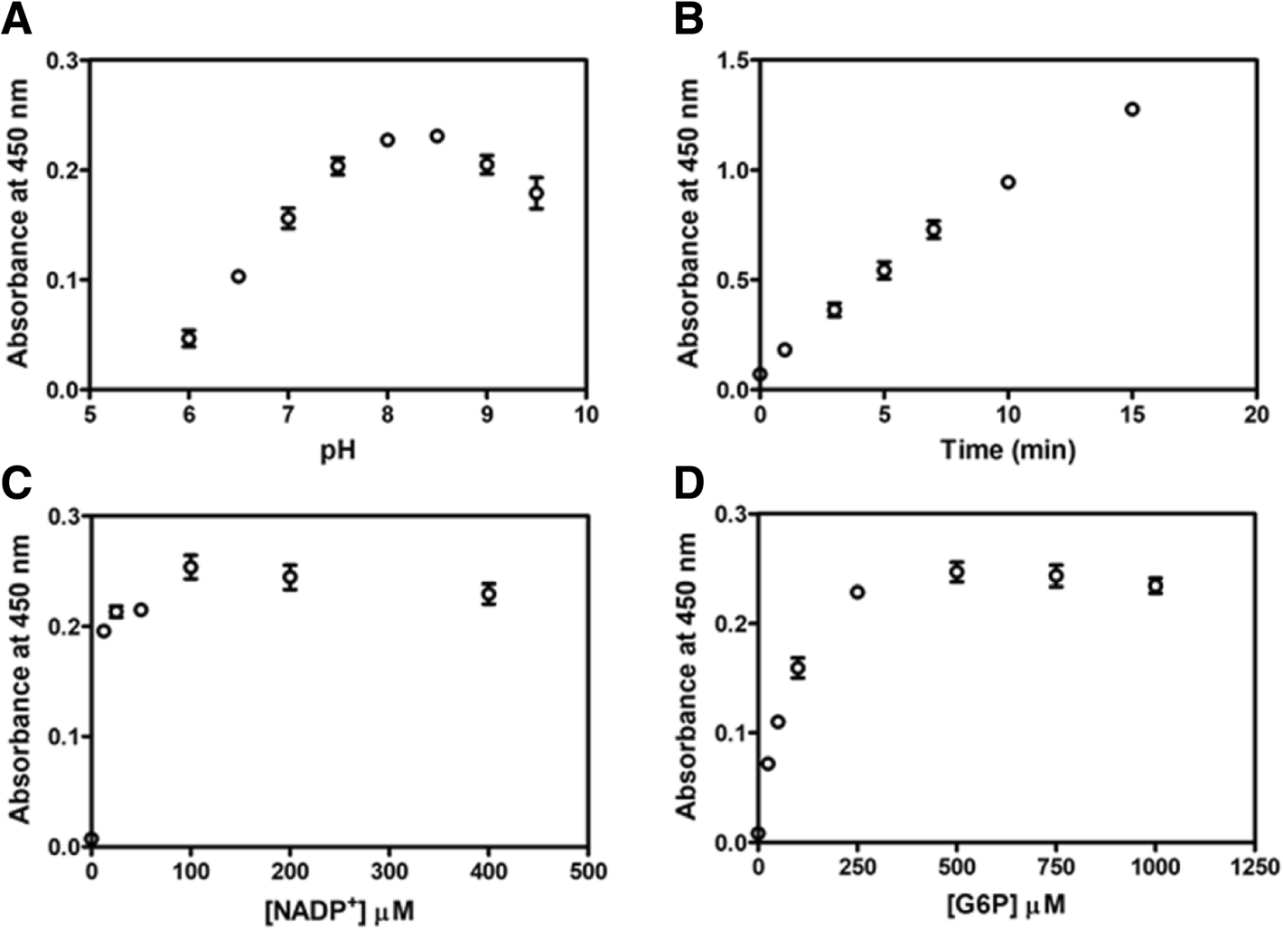
Optimization of G6PD assay conditions using colorimetric WST-8. **a** The effect of pH on enzymatic reaction. **b** Reaction time course of the assay. **c** The effect of NADP^+^ concentration on the enzymatic assay. **d** The effect of G6P concentration on the enzymatic assay

For the human G6PD assay using WST-8, similar parameters were optimized as described for GDH, except that NADP^+^ and G6P were used instead of NAD^+^ and glucose, respectively. The optimum pH for the G6PD assay was observed at a pH of around 8.0 and this pH value was used for further experiments (Fig. 2a). The optimum pH obtained from WST-8 assay agrees with previous studies where G6PD activity was determined using UV-spectrophotometry, in which NAD(P)H absorption at 340 nm was measured [32, 36, 37]. To determine the adequate incubation time for the G6PD activity assay, the progress of the reaction was followed by measuring absorbance at 450 nm for 15 min. A linear correlation of formazan product formation was observed with incubation time up to 10 min (Fig. 2b). There was no significant difference in absorbance at 450 nm using NADP^+^ concentrations from 12.5 to 400 μM in the assay (Fig. 2c). Absorbance at 450 nm reached a plateau when assayed in the presence of 250 μM G6P (Fig. 2d). Hence, to provide sufficient WST-8 and substrates, a G6PD activity assay using WST-8 was monitored in the presence of 500 μM G6P and 200 μM NADP^+^ for 10 min in further experiments.

### Comparison of colorimetric tetrazolium-based assay with the standard UV-spectrophotometric method

#### Linearity and the limit of detection

**Fig. 3:**
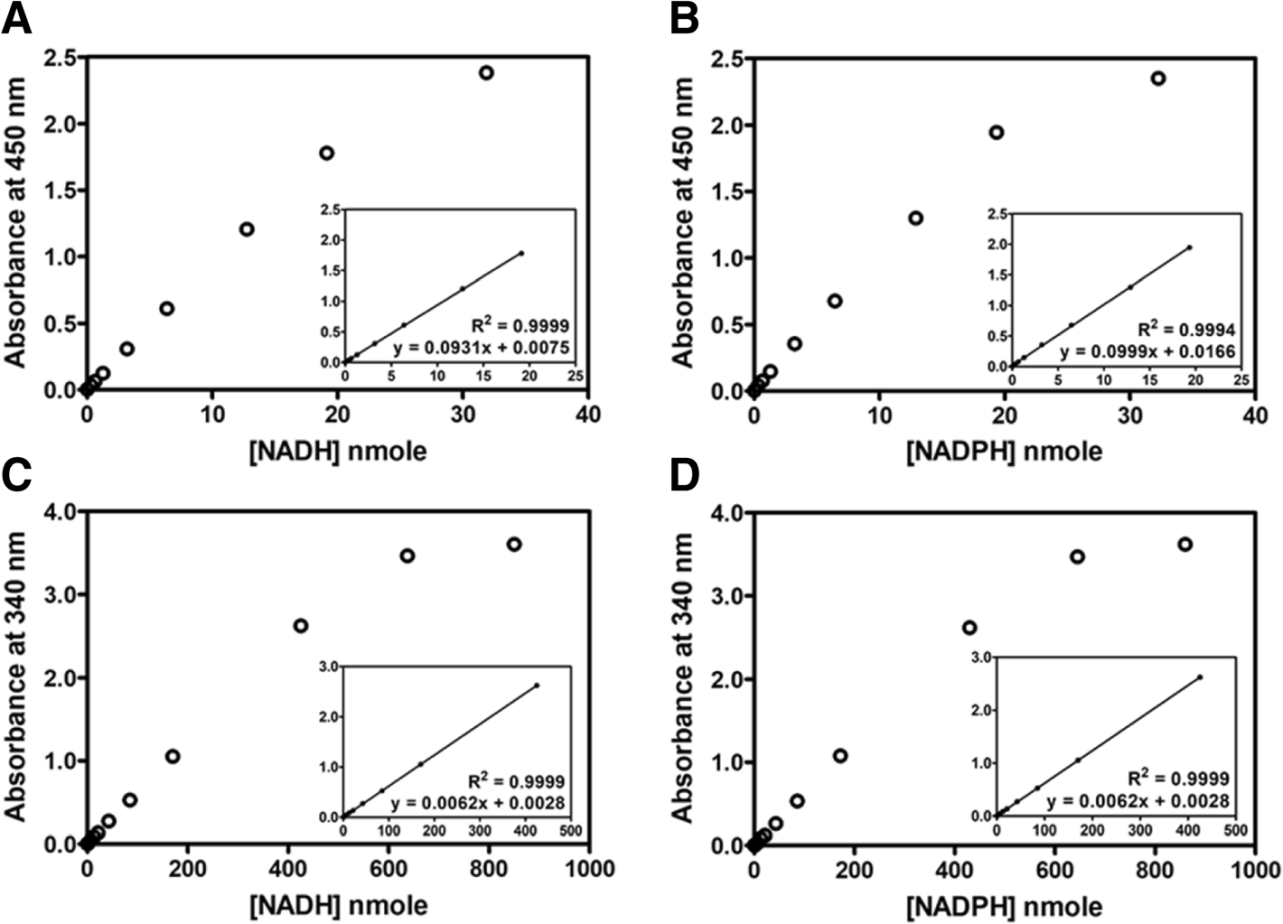
NADH and NADPH standard curves. **a** NADH was measured using colorimetric WST-8. **b** NADPH was measured using colorimetric WST-8. **c** NADH was measured at 340 nm using UV-spectrophotometry. **d** NADPH was measured at 340 nm using UV-spectrophotometry. Insets demonstrate assay linearity over the range of concentrations 0 to 19 nmole of NAD(P) measured by colorimetric WST-8 assay and 0 to 400 nmole of NAD(P)H measured by UV-spectrophotometry

Under the optimized conditions, the limit of detection was determined for the colorimetric WST-8 assay and UV-spectrophotometry. The standard curves of NAD(P)H were constructed for the two methods by plotting absorbance against NAD(P)H concentration. For the WST-8 assay, a good linear correlation between absorbance at 450 nm and amount of NAD(P)H was obtained in the range of 0–19 nmole, with R^2^ values > 0.99 (Fig. 3a and b). For the UV-spectrophotometric method, the plot of absorbance at 340 nm versus the amount of NAD(P)H also showed a linear response, with a good correlation coefficient (R^2^ values > 0.99), but with a wider assay window of NAD(P)H amounts ranging from 0 to 400 nmole (Fig. 3c and d).

**Fig. 4:**
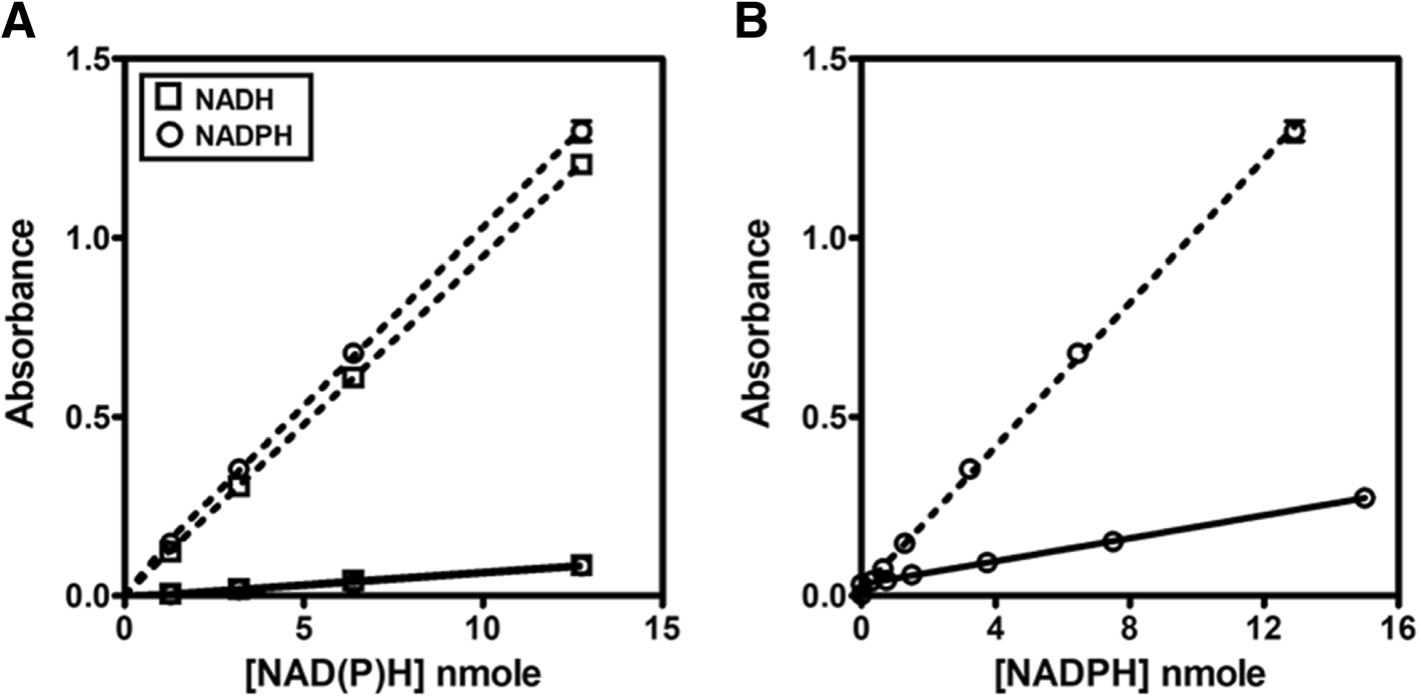
The relationship between absorbance and NAD(P)H concentration. NAD(P)H was measured using colorimetric WST-8 at 450 nm (dashed line) and UV-spectrophotometry at 340 nm (solid line). Measurement of NAD(P)H at 340 nm using UV-spectrophotometry was performed in **a** 1 mL cuvette or **b** 96-well plate

The limit of detection (LOD) and lower limit of quantitation (LLOQ) of the assay were calculated as the sample blank value plus three and ten standard deviations, respectively (Table 1). The colorimetric WST-8 assay exhibited greater sensitivity than the UV-spectrophotometric method for the detection of NAD(P)H. In terms of detection limit, WST-8 could measure as low as 0.32 and 0.29 nmole of NADH and NADPH, respectively. This is approximately 5-fold more sensitive than UV-spectrophotometry, which measures NAD(P)H absorption at 340 nm, where it could detect 1.65 and 1.72 nmole of NADH and NADPH, respectively. The LLOQ and upper limit of quantitation (ULOQ) of NAD(P)H using the WST-8 method is 0.4 and 19 nmole, respectively. The LLOQ of the WST-8 assay (0.4 nmole) was about 10-fold lower than that determined by UV-spectrophotometry (3.41 nmole), suggesting greater sensitivity of NAD(P)H detection by WST-8 method. However, UV-spectrophotometry exhibited a longer linear response of NAD(P)H (0–400 nmole) than the colorimetric WST-8 assay. The ULOQ for UV-spectrophotometry is 400 nmole. This indicates a superior NAD(P)H detection range by UV-spectrophotometric method. Figure 4 shows the linear response of WST-8 for NAD(P)H detection, compared with UV-spectrophotometry. A comparison between WST-8 and UV-spectrophotometry was demonstrated. The WST-8 based assay showed greater sensitivity (approximately 5-fold), compared with measurement of NAD(P)H at 340 nm using UV-spectrophotometry in a 1 mL cuvette (Fig. 4a). This agrees well with a direct comparison between WST-8 and UV-spectrophotometry in a 96-well format (Fig. 4b), suggesting that the WST-8 assay is suitable for high-throughput screening.

**Table 1:** The limit of NAD(P)H detection of colorimetric WST-8 and UV spectrophotometric methods

Method		LOD (nmole)	LLOQ (nmole)	ULOQ (nmole)
Colorimetric WST-8 (450 nm)	NADH	0.32	0.4	19
	NADPH	0.29	0.4	19
UV spectrophotometry (340 nm)	NADH	1.65	3.41	400
	NADPH	1.72	4.11	400

#### Accuracy and precision

To determine the repeatability, precision and accuracy of colorimetric WST-8 and UV-spectrophotometric methods, three different NAD(P)H amounts (4–13 nmole) within the standard range were measured on five sequential days in triplicate. The accuracy and precision of the WST-8-based assay for measuring NAD(P)H are shown in Table 2. In terms of accuracy and precision, the performance of the WST-8 assay for NAD(P)H detection was comparable to standard UV-spectrophotometry, which measures absorbance at 340 nm of NAD(P)H. The measured NAD(P)H amount using WST-8 assay are very close to the prepared amount, giving only small numbers (0.7–2.5) of %RE that have fallen within the statistical acceptance criteria for analytical run (15%) [40, 41]. In addition, both the within-run and between-run precisions of the WST-8 assay are also within (0.6–4.5), the statistical acceptance criteria (15%) [40, 41]. These values were indistinguishable from the UV-spectrophotometric method, which measures absorbance at 340 nm, suggesting that the WST-8 assay can be used in place of the UV-spectrophotometric method to measure NAD(P)H.

**Table 2:** Accuracy and precision for measurement of NAD(P)H by colorimetric WST-8 and UV spectrophotometry methods

Method		Prepared amount (nmole)	Measured amount (nmole)	Accuracy (%RE)	Precision (%CV)		Z’- factor
					Within-run	Between-run	
Colorimetric WST-8 (450 nm)	NADH	4.3	4.33	1.9	1.8	1.7	0.93
		8.5	8.44	−0.7	1.3	1.0	0.95
		12.8	12.44	−2.5	0.6	0.9	0.98
	NADPH	4.3	4.39	2.1	2.2	4.5	0.90
		8.6	8.72	1.4	0.8	1.7	0.99
		12.9	12.98	0.6	1.5	2.1	0.98
UV-spectrophotometry (340 nm)	NADH	102	101.70	−0.3	0.7	1.2	0.97
		212.5	208.61	−1.8	1.1	1.2	0.96
		340	329.34	−3.1	1.1	1.0	0.96
	NADPH	103.2	105.50	2.2	1.0	1.1	0.96
		215	215.88	0.4	1.3	0.8	0.96
		344	344.91	0.3	0.5	0.9	0.98

Furthermore, the robustness of colorimetric WST-8 for high-throughput screening was evaluated by determining the Z’ factor. The means and standard deviations of the negative control were used to calculate the Z’ factor according to the equation developed by Zhang and colleagues [42]. Z’ factor is defined as Z’ = 1 – [(3σ_pos_ + 3σ_neg_)/|μ_pos_ - μ_neg_|]. It is calculated based on the scale of the assay and the distribution of the positive and negative signals, and has a value between 0 and 1. An assay with a wide separation, i.e. close to 1, would be a good assay. WST-8 exhibited excellent reproducibility, with Z’ values ranging between 0.9 to 0.99 for NAD(P)H measurement, which are comparable to UV-spectrophotometry (0.96–0.98). The Z’ factor of WST-8 was greater than the analytical acceptance criteria (0.7), indicating that the WST-8 assay is suitable for high-throughput screening.

### Assay interference of WST-8/1-mPMS system

**Fig. 5:**
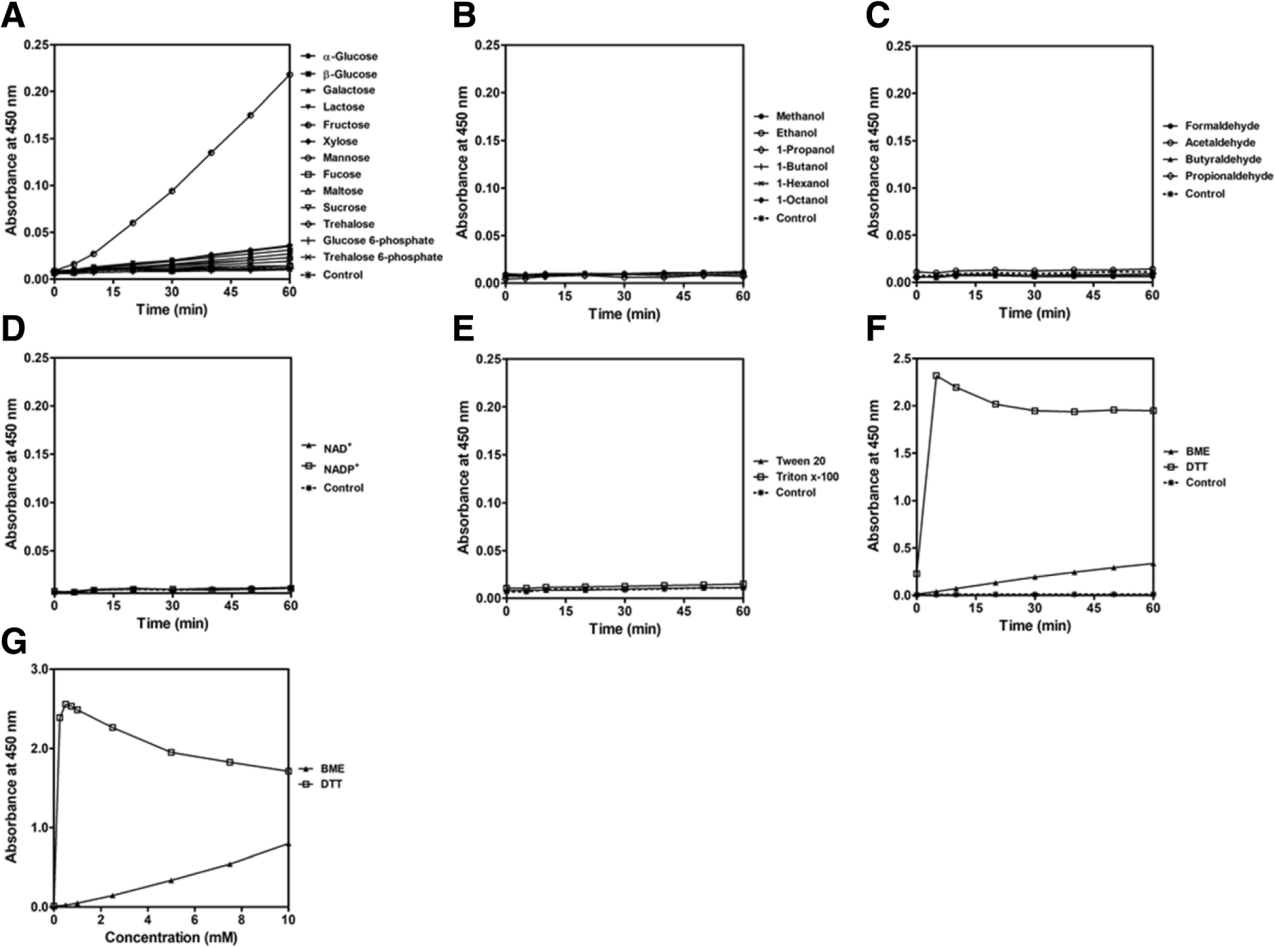
The effect of various chemicals on the reduction of WST-8. Background absorbance in the absence of NAD(P)H of **a** sugars, **b** alcohols, **c** aldehydes, **d** NAD(P)^+^, **e** detergents, **f** reducing agents and **g** dose-response curve of reducing agents

As WST-8 is reduced to form the formazan product, which can be measured at 450 nm, the presence of other chemicals that can reduce WST-8 may cause a false positive reading in the assay. Various dehydrogenase substrates (sugars, alcohols and aldehydes), common detergents (triton x-100 and tween 20) and reducing agents (β-mercaptoethanol (BME) and dithiothreitol (DTT)) were tested for their ability to reduce WST-8 in the absence of NAD(P)H. Interestingly, among various sugars tested in this study, fructose potentially reduced WST-8 and produced formazan product, causing a false-positive reading (Fig. 5a), whereas alcohols and aldehydes showed no interference in the WST-8/1-mPMS detection system (Fig. 5b and c). Likewise, the oxidized forms of cofactors (NAD^+^ and NADP^+^) and common detergents (up to 1%) did not interfere with the reduction of WST-8 (Fig. 5d and e). Reducing agents significantly reduced WST-8 to form the formazan product in the absence of NAD(P)H, in which DTT exhibited a stronger reducing capacity than BME (Fig. 5f). A dose-response curve of both reducing agents illustrated that as low as 0.25 mM DTT could cause a false positive reading in the WST-8/1-mPMS detection system (Fig. 5g). The reduction ability of BME on WST-8 has also been reported previously [43].

### Dehydrogenase activity assay

WST-8 was applied to measure GDH and G6PD activities in different sample types—purified enzymes and crude extracts containing the enzyme. The dehydrogenase activities of purified *Bacillus* GDH and human G6PD enzymes were comparable to those obtained from conventional UV-spectrophotometry (Table 3). The activities of purified GDH were 0.069 ± 0.001 and 0.078 ± 0.004 nmole/min/μg by colorimetric WST-8 and UV-spectrophotometric methods, respectively. For purified G6PD, the activities of 12.51 ± 0.36 and 14.88 ± 0.39 nmole/min/μg were determined by WST-8 and UV-spectrophotometric methods, respectively. The differences were 15%, though the value obtained from WST-8 was slightly lower.

**Table 3:** GDH and G6PD activities measured by colorimetric WST-8 and UV spectrophotometry methods

Method	Enzyme activity (nmole/min/μg)		
	Purified GDH	Purified G6PD	G6PD in crude extract
Colorimetric WST-8 (450 nm)	0.069 ± 0.001	12.51 ± 0.36	0.112 ± 0.001
UV-spectrophotometry (340 nm)	0.078 ± 0.004	14.88 ± 0.39	0.145 ± 0.003

The performance of WST-8 for measuring dehydrogenase activity in biological samples was assessed using *E. coli* crude extract. The recombinant human G6PD activity of 0.112 ± 0.001 nmole/min/μg was obtained, comparable to that of UV-spectrophotometry, 0.145 ± 0.003 nmole/min/μg. This indicates that WST-8 is a sensitive assay for the detection of dehydrogenase enzyme activity. It also suggests that the colorimetric WST-8 assay can measure dehydrogenase activity in biological samples that usually contain several other proteins and enzymes.

To evaluate the performance of the colorimetric WST-8 assay, a spike recovery was carried out for both GDH and G6PD (Table 4). Known amounts of GDH (1 μg) and G6PD (0.2 μg) were spiked into FBS and enzyme activity was monitored by measuring absorbance at 450 nm. Percent recovery of 118 and 120 was obtained for GDH and G6PD, respectively, which is within the statistical acceptance criteria (80–120%) [44]. It was, again, demonstrated here that the colorimetric WST-8 assay satisfactorily recovered GDH and G6PD in the FBS sample.

**Table 4:** Spike recoveries of GDH and G6PD in FBS using colorimetric WST-8 assay

Assay	Enzyme activity (nmole/min)		%Recovery
	Expected	Measured	
GDH	0.071	0.084 ± 0.002	118.78 ± 1.81
G6PD	1.024	1.235 ± 0.004	120.57 ± 0.33

**Fig. 6:**
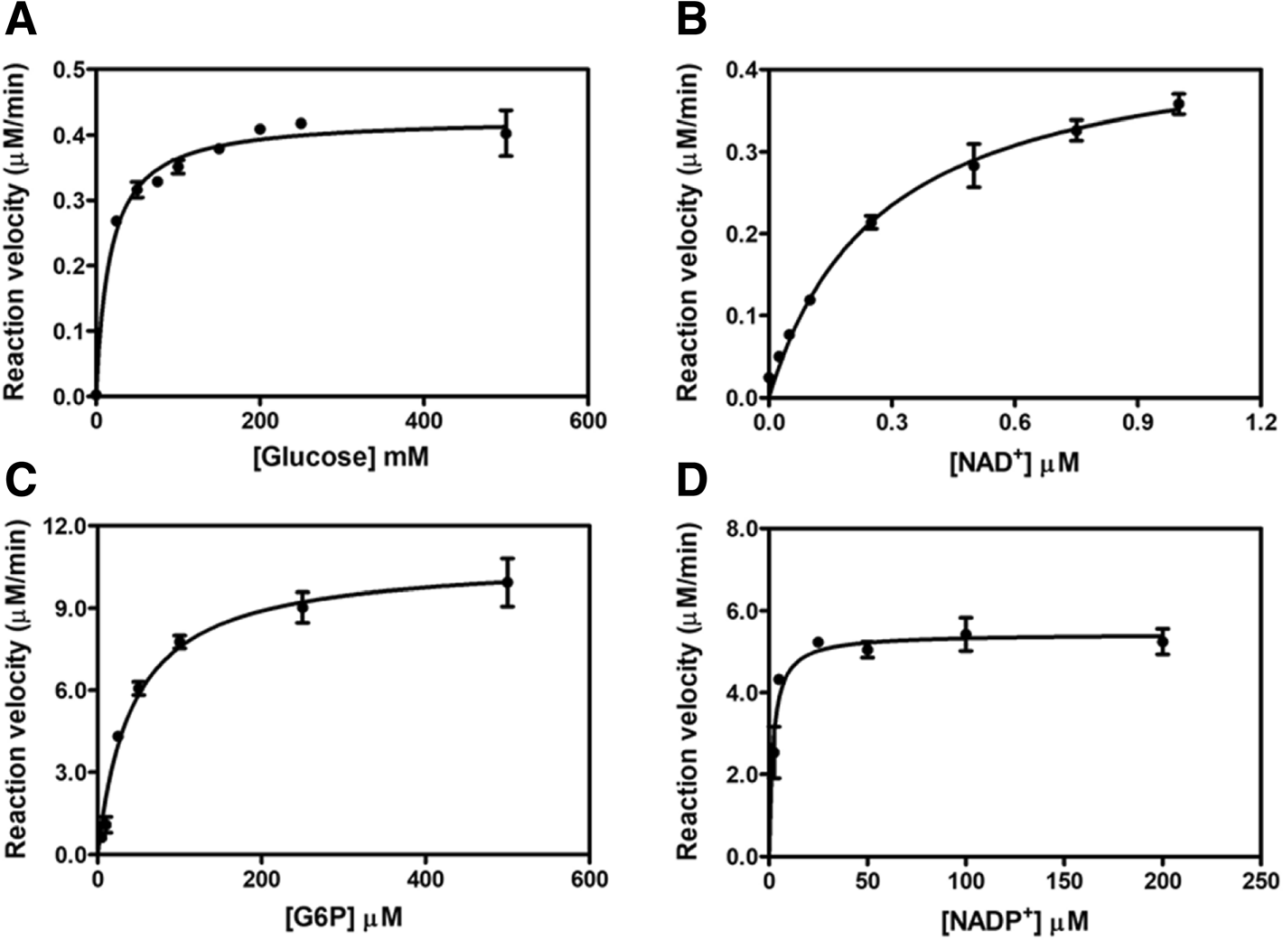
Steady-state kinetics assay determined by colorimetric WST-8 method. Enzyme kinetic plots of GDH for **a** glucose and **b** NAD^+^. Enzyme kinetic plots of G6PD for **c** G6P and **d** NADP^+^

To further assess the reliability of the WST-8 assay, steady-state kinetic parameters were determined for both GDH and G6PD. The Michaelis-Menten plots of GDH and G6PD are shown in Fig. 6. Michaelis-Menten constants were obtained by nonlinear regression fitting of the Michaelis-Menten equation and the kinetic parameters obtained for GDH and G6PD determined by WST-8 method are shown in Table 5. The *K*_*M*_ values for glucose (17.1 ± 2.0 mM) and NAD^+^ (0.271 ± 0.027 mM) were determined for GDH. Likewise, the *K*_*M*_ values for G6P (42.3 ± 4.8 μM) and NADP^+^ (2.1 ± 0.4 μM) were obtained for G6PD. These steady-state kinetic parameters were comparable to those reported previously for UV-spectrophotometry [32, 35–37].

**Table 5:** Steady-state kinetic parameters of GDH and G6PD determined by colorimetric WST-8 assay compared with previous studies using UV-spectrophotometry

Enzyme	*K*_*M*_ (mM)		Reference
	Glucose	NAD^+^	
*Bacillus* GDH	17.1 ± 2.0	0.271 ± 0.027	The present study
	31.8 ± 0.4	0.210 ± 0.001	Chen et al., 2011 [35]
Enzyme	*K*_*M*_ (μM)		References
	G6P	NADP^+^	
Human G6PD	42.3 ± 4.8	2.1 ± 0.4	The present study
	47.8 ± 4.2	7.2 ± 1.8	Boonyuen et al., 2016 [32]
	45.8 ± 1.6	4.67 ± 0.32	Huang et al., 2008 [36]
	52 ± 4	7.07 ± 1.13	Wang et al., 2005 [37]

### Application of WST-8 based assay for substrate screening of an uncharacterized SDR from *B. pseudomallei*

Previously, we could detect the GDH activity of an uncharacterized SDR from *B. pseudomallei* using a commercial GDH activity assay kit (Biovision, Mountain View, USA) [45]; however, the true function of this protein is still unknown. Proteins in the SDR family show a broad substrate specificity towards sugars, alcohols, aldehydes, ketones and fatty acids. Due to the excellent sensitivity of the WST-8-based assay developed, the method was applied to screen for SDR substrate from *B. pseudomallei* (Table 6). The crude lysate of *E. coli* expressing SDR showed dehydrogenase activity towards ethanol and 1-propanol with NAD^+^ as a cofactor. In addition, with NADP^+^ as a cofactor, dehydrogenase activity of uncharacterized SDR was observed for glucose, xylose, butanol, hexanol and octanol, while no detectable activity was seen for aldehyde substrates. Previously, several SDR enzymes have been shown to have activity towards a wide variety of substrates. Gox2036, an SDR from *Gluconobacter oxydans*, exhibited oxidative activity against several alcohols and diols [46]. SDR from Bacillus sp. ZJ showed remarkable catalytic activity in the oxidation of glucose, suggesting its function as glucose dehydrogenase. However, BzGDH also showed activity toward various sugars, such as galactose, mannose, xylose, maltose, and lactose [47]. Currently, the SDR from *B. pseudomallei* is being purified and characterized to confirm the genuine function of this putative protein.

**Table 6:** Substrate screening of crude extract of *E. coli* harboring SDR from *B. pseudomallei*

Substrate	Enzyme activity (nM/min)	
	NAD^+^	NADP^+^
Sugars		
D-Glucose	na	158.67 ± 24.36
D-Galactose	na	na
D-Lactose	na	na
D-Fructose	na	na
D-Xylose	na	25.89 ± 14.14
D-Mannose	na	na
Maltose	na	na
Sucrose	na	na
Alcohols and aldehydes		
Methanol	na	na
Ethanol	131.31 ± 25.26	na
1-Propanol	145.60 ± 8.42	na
1-Butanol	na	134.23 ± 16.50
1-Hexanol	na	90.34 ± 4.72
1-Octanol	na	33.11 ± 11.78
Acetaldehyde	na	na
Propionaldehyde	na	na
Butyraldehyde	na	na

*na* no activity

## Discussion

In this study, we described the application of tetrazolium salt WST-8 to determine dehydrogenase activity using GDH and G6PD as model enzymes. The reaction catalyzed by GDH generates NADH, while that catalyzed by G6PD produces NADPH. In the presence of 1-mPMS, an electron mediator, NAD(P)H reacts with WST-8 to yield a formazan product, which can be determined by measuring absorbance at 450 nm. The assay is applicable to the quantitative measurement of NAD(P)H produced from dehydrogenase reactions. Moreover, the WST-8 assay developed was applied to screening the substrate of an uncharacterized SDR from *B. pseudomallei.* The results indicated that the colorimetric WST-8 method has several advantages for measuring NAD(P)H and dehydrogenase activity. The method shows great sensitivity in the detection of NAD(P)H, where as low as 0.3 nmole can be detected. It is 5-fold more sensitive than conventional UV-spectrophotometry, which measures NAD(P)H absorption at 340 nm. The greater sensitivity of the WST-8 assay when compared to UV-spectrophotometry can be explained by a higher extinction coefficient of formazan product (30,700 M^− 1^·cm^− 1^), which is about 5-fold greater than that of NAD(P)H (6,220 M^− 1^·cm^− 1^) [21]. Although UV-spectrophotometry is able to measure small amounts of NAD(P)H accurately (i.e. as low as 1.7 nmole), the technique is slow and is, therefore, not suitable for high-throughput dehydrogenase-activity screening. On the other hand, the colorimetric WST-8 assay can be performed in a 96-well plate with a final volume of 150 μl and the volume can further be minimized for the purposes of economy. Though NAD(P)H absorption at 340 nm can also be measured in a 96-well plate, the lower extinction coefficient of NAD(P)H and small reaction volume usually give low absorbance. This makes conventional UV-spectrometry less sensitive than colorimetric WST-8 assay.

It is worth mentioning that NAD(P)H detection method could be chosen according to the quantity of the NAD(P)H. Although WST-8 is highly sensitive and uses a low detection amount of NAD(P)H, its linear range is much smaller than other NAD(P)H detection methods. Therefore, serial dilutions are necessary when a large amount of NAD(P)H is present, to ensure that the detection value is within the linearity range of the WST-8 method (0.4–19 nmole). In contrast, the UV-spectrophotometric method is more appropriate to measure a high amount of NAD(P)H (i.e. > 1.72 nmole). The physiological concentration of NAD(P)H varies among cell types. The concentration of NADH is approximately 100 μM (15 nmole in 150 μL reaction) and 60 μM (10 nmole in 150 μL reaction) in breast and brain tissues, respectively [48, 49]. In bacterial cells, the NADH concentration is 4–11.7 μmole/g [50]. The WST-8 method could be used to measure the amount of NAD(P)H in all of these biological samples, because the concentrations present in the samples are within the detection limit of the assay.

Dehydrogenase activity measurement using colorimetric WST-8 is an end-point assay and could be more time-consuming than conventional UV-spectrophotometry, because the WST-8 assay usually requires a long incubation time. However, the required incubation time in the WST-8 assay depends upon enzyme activity. In this study, we monitored GDH activity at 60 min and G6PD activity at 10 min to ensure maximum sensitivity. In fact, the enzyme activity of GDH and G6PD can be determined using a shorter incubation time; any time points that are in the linear absorbance range can be used. Nevertheless, the shortest incubation time should give measurable absorbance and this value should be greater than the detection limit of a spectrophotometer. Our results indicate that dehydrogenase activity can be measured at 5 min for GDH and 1 min for G6PD, which is similar to those monitored by UV-spectrophotometry.

In terms of accuracy, precision and reproducibility, the performance of the colorimetric WST-8 assay is indistinguishable from UV-spectrometric method that measures the absorbance of NAD(P)H at 340 nm. The WST-8 method shows high accuracy and precision for measuring NAD(P)H. For accuracy, the method exhibited %RE of 0.7–0.25. For precision, %CV for within-run and between-run ranged between 0.6–4.5. These values were very small and also within the statistical acceptance criteria of 15%. Excellent reproducibility, with Z’ values of 0.9–0.99, was observed for the WST-8/1-mPMS detection system. Altogether, this suggests that the WST-8 assay is suitable for high-throughput screening in clinical and research applications. In addition, it was demonstrated here that the WST-8 assay is effective for measuring the dehydrogenase activities of purified enzymes as well as those of biological samples. Dehydrogenase activity measured using WST-8 was comparable to that obtained from UV-spectrophotometry. Though enzyme activity measured by WST-8 was 10–25% lower than that measured by UV spectrophotometry, it was not statistically significant by Mann-Whitney analysis. *p*-values of 0.0765, 0.1 and 0.0765 were obtained for activity measurement of purified GDH, purified G6PD, and crude G6PD assay, respectively. This indicates that the WST-8 method is accurate and also compatible with dehydrogenase activity measurement in the presence of other contaminated proteins. Even though WST-8 is quite stable and is not light-sensitive, the reagent should be kept in the dark and exposure to the air avoided, to minimize any decrease in its activity. More importantly, the developed method was further applied for substrate screening of an uncharacterized SDR from *B. pseudomallei*, in which the frequently used UV-spectrophotometric method is not precisely responsive*.* In fact, no dehydrogenase activity was observed by direct NAD(P)H absorbance measurement. On the other hand, dehydrogenase activity was detected for glucose, xylose, ethanol, propanol, butanol, hexanol, and octanol, when assayed using the WST-8 method. This indicates that the WST-8 based assay is essential for measuring low concentrations of NAD(P)H. The WST-8 assay is sensitive and able to measure the dehydrogenase activities of uncharacterized SDR in bacterial crude lysate. Work is currently underway to investigate the true function of uncharacterized SDR from *B. pseudomallei*.

It was reported that the UV-spectrophotometric method was not adequately sensitive to detect hydroxysteroid dehydrogenase activity, since it is often present at low levels, especially in biological samples. Therefore, a greater sensitivity (2–3 fold) nitroblue tetrazolium salt was used to detect activity in steroidogenic tissue extracts [51]. In addition to sensitivity, direct measurement of NAD(P)H at 340 nm from lysate samples usually results in low absorbance, of around 0.04–0.05, leading to high noise/signal ratio in the lysate assay [52].

Compared with other qualitative and quantitative dehydrogenase kits that must be stored at − 20 °C and are stable for only 2–3 months, the WST-8 solution is highly stable and can be stored at 4 °C for up to 1 year (according to the manufacturer). Though measurement using WST-8 assay requires reagent preparation, it is easy and the cost per reaction is much lower ($0.35) than commercial dehydrogenase assay kits ($3.50). According to the results shown here, monosaccharide and disaccharide sugars, alcohols, aldehydes and detergents did not interfere with the current developed method. However, it should be cautioned that the WST-8/1-mPMS detection system is not compatible with a reducing agent in buffer systems, as this can generate false-positive signals. In addition, caution should be taken when working with ketonic monosaccharides, such as fructose. On the other hand, some chemicals may interfere with the measurement of NAD(P)H at 340 nm, including bilirubin and several inorganic compounds, such as phosphorus, nickel, and chromium [53]. Furthermore, hemoglobin also interferes with the spectrophotometric detection of NAD(P)H at 340 nm, causing a shift in the UV spectra [54]. Both bilirubin and hemoglobin are endogenous substances in many biological samples. Therefore, it should be taken into consideration when using the UV method to measure dehydrogenase in biological samples directly.

## Conclusions

WST-8 is a sensitive assay for the measurement of NAD(P)H and dehydrogenase activity. The method is specific for NAD(P)H and measurement of dehydrogenase activity. In addition to sensitivity, the lower cost per reaction, high stability and relative ease of preparation and long-term storage life are other advantages of the WST-8 assay. The assay could serve as a high-throughput method for measuring dehydrogenase activity.

## Abbreviations

%CV: Percent coefficient of variation
%RE: Percent relative error
1-mPMS: 1-methoxy-5-methylphenazium methylsulfate
BME: β-mercaptoethanol
DTT: Dithiothreitol
G6P: Glucose-6-phosphate
G6PD: Glucose-6-phosphate dehydrogenase
GDH: Glucose dehydrogenase
IPTG: Isopropyl β-D-thiogalactoside
LLOQ: Lower limit of quantitation
LOD: Limit of detection
NAD^+^: Oxidized form of nicotinamide adenine dinucleotide
NADH: Reduced form of nicotinamide adenine dinucleotide
NADPH: Reduced form of nicotinamide adenine dinucleotide phosphate
NAPD^+^: Oxidized form of nicotinamide adenine dinucleotide phosphate
SDR: Short-chain dehydrogenase/oxidoreductase
SDS-PAGE: Sodium dodecyl sulfate-polyacrylamide gel electrophoresis
ULOQ: Upper limit of quantitation
WST: Water-soluble tetrazolium salts
